# Gender and Racial Discrimination During Residency Training: Scoping Review

**DOI:** 10.2196/87524

**Published:** 2026-04-02

**Authors:** Ida John, Liz Dennett, Julie Nguyen, Marghalara Rashid

**Affiliations:** 1 Department of Pediatrics, Faculty of Medicine and Dentistry, University of Alberta, 3-591 Dianne and Irving Kipnes Health Research Academy, 11405 87 Avenue NW, Edmonton, AB, T6G 1C9, Canada, 1 780 248 5582; 2 Geoffrey and Robyn Sperber Health Sciences Library, University of Alberta, Edmonton, AB, Canada

**Keywords:** discrimination, gender, racial, residents, scoping review

## Abstract

**Background:**

Women and visible minorities (VMs) continue to face discrimination while working in health care. These instances of discrimination can range from those perpetrated by individuals, such as misidentification, to prejudices held by institutions, such as a lack of commitment to hiring VMs. Furthermore, many residents face unique experiences of discrimination due to the intersection of gender and race. Although numerous studies have been published on the experiences of physicians with discrimination, there is a limited literature specifically on the experiences of residents.

**Objective:**

This study aimed to explore and analyze the impact of gender and racial discrimination on medical residents during their residency training.

**Methods:**

This scoping review was conducted in accordance with the PRISMA-ScR (Preferred Reporting Items for Systematic Reviews and Meta-Analyses Extension for Scoping Reviews) guidelines. A health sciences librarian searched 5 databases: Ovid Medline(R) ALL, Embase (via Ovid), APA PsycInfo (via Ovid), CINAHL Plus with Full Text (via EBSCOhost), and Scopus. No study design, country, or date restrictions were applied. Studies that explored the impact of both gender and racial discrimination on residents during their residency training were included. Two reviewers conducted title and abstract screening, followed by full-text screening, and any discrepancies were resolved through group consensus. This study’s content was extracted using Microsoft Word to create tables for organizing and managing the information from the included studies.

**Results:**

After removing duplicates, the literature search revealed 2435 papers for title and abstract screening. A total of 340 papers were selected for full-text review, and ultimately, 26 papers met the inclusion criteria. Included papers were published between 1995 and 2024 in the United States (n=16), Canada (n=4), Australia and New Zealand (n=2), Saudi Arabia (n=1), Mexico (n=1), South Africa (n=1), and the United Kingdom (n=1). Our analysis revealed four themes: (1) forms of discrimination faced by VMs, (2) sources of discrimination, (3) ramifications of discrimination, and (4) ways for individuals and institutions to reduce gender and racial discrimination.

**Conclusions:**

This scoping review identified that discrimination primarily manifests as microaggressions against residents and revealed the negative impact it continues to have on their careers. Raising awareness about these issues can help programs and institutions develop tailored solutions to tackle these problems and provide a safe, inclusive training environment for all residents.

## Introduction

### Background

Gender and racial discrimination continue to be prevalent across society, especially within occupational settings, where most people report experiencing discrimination [1]. Discrimination refers to an “action, behavior, decision, or omission that treats a person or a group of people unfairly” due to traits such as race or gender [2]. These biases can be explicit or implicit but are more often implicit, as reported in the current literature [3,4]. Additionally, these instances of discrimination can range from occurring within individual interactions to institutional policies that marginalize groups of people, such as women and visible minorities (VMs) [5]. VMs refer to “persons, other than Aboriginal peoples, who are non-White in color” [6]. Within health care, many studies surveying physicians have found that women physicians and physicians of VM status report higher rates of patients refusing their care due to their race and/or gender [7,8]. Both women and VMs are also more likely to be mistaken for nurses [8] or as maintenance and housekeeping staff [9]. In addition to these forms of discrimination, some individuals face prejudice stemming from the intersection of their gender and race, known as intersectionality [10]. Intersectionality is defined as “the interaction between gender, race, and other categories of difference in individual lives, social practices, institutional arrangements, and cultural ideologies, and the outcomes of these interactions in terms of power” [11]. An example of what intersectional discrimination could look like within health care can be seen in a study conducted by Pololi and Jones [12], where they interviewed women who were medical faculty regarding their experiences. Women of VM status reported how their double minority status impacted them in the workplace, especially how isolating it felt to be the only woman and/or person of VM status on the faculty [12].

Medical residents hold a unique position within health care, as they are simultaneously trainees and practitioners interacting with and caring for patients under supervision. Recent research conducted by Mocanu et al [13] surveyed general surgery residents on the effects that their gender and/or VM status had on their resilience and their residency training experience. Some experiences of discrimination reported were similar to those reported by physicians, such as how the majority of women residents and residents of VM status faced dismissal of their medical advice by patients due to their gender or race [13]. Residents also believed that they received fewer training opportunities due to their gender and/or race [13]. Another study conducted by Bhatt [14] found that women or men of VM status were rarely selected as chief residents; often, they were White men, even though residents are a diverse group. Residents also described being doubly discriminated against due to their gender and race [14].

Although individual research studies have been conducted on the experiences of residents facing discrimination, to the best of our knowledge, this is the first paper that compiles resident experiences of discrimination into 1 paper. This scoping review aims to explore and analyze existing literature on the impact of gender and racial discrimination on residents.

### Positionality Statement

We acknowledge that our perspectives and identities may have influenced our scoping review. However, through reflexivity and debriefings as a team, we have examined potential preconceived assumptions and strived for an accurate and rigorous representation of the existing literature. The team includes diverse members. IJ is a woman from a VM group and a graduate research assistant on the project. LD is a White female librarian who conducted a comprehensive search for this scoping review. JN is from a VM background and is a research assistant. MR is a VM woman and a scholar in medical education whose work focuses on the experiences of structurally marginalized learners.

## Methods

### Overview

We used the Arksey and O’Malley [15] framework, which consists of 5 steps: identifying the research question, finding relevant reports, selecting reports, extracting data, and summarizing the results. Furthermore, for our data reporting, the PRISMA-ScR (Preferred Reporting Items for Systematic Reviews and Meta-Analyses Extension for Scoping Reviews) guidelines were used to ensure transparent and clear reporting of the included studies in our scoping review [16]. Additionally, the protocol for this scoping review was developed in accordance with these guidelines [16]. The protocol has not been registered.

### Study Eligibility Criteria

Included studies focused on medical residents who faced both gender and racial discrimination during their residency training. We included peer-reviewed studies, books, book chapters, peer-reviewed abstracts, dissertations, and papers written in English. Papers that explored the experiences of other populations, such as undergraduate medical students, fellows, clinical and academic faculty, health care professionals, or allied health care professionals, were excluded. Papers that did not align with our study objectives were excluded. Studies that focused solely on collecting data on gender or racial discrimination were not included, as the purpose of this scoping review was to explore the experiences of residents who faced discrimination based on both gender and race. Literature surrounding the impact of discrimination on resident assessments and evaluations was excluded, as there are abundant studies and reviews regarding this specific topic. We also removed studies focused on sexual orientation, as it is not the focus of this review and is a large topic in its own right. Studies that were not conducted in a residency setting were excluded. We also excluded the following: policy documents, vlogs, websites, working papers, newsletters, government publications, videos, study protocols, and papers written in other languages. There were no limitations imposed on the study design, the time frame, or the countries where the research was conducted.

### Search Strategy

A health sciences librarian (LD) and other research team members collaborated to develop an initial search strategy to identify studies on the experiences of medical residents with intersectional gender and racial discrimination. The health sciences librarian then ran the search in the following databases: Ovid Medline(R) ALL, Embase (via Ovid), APA PsycInfo (via Ovid), CINAHL Plus with Full Text (via EBSCOhost), and Scopus from inception until May 2, 2025. The strategy combined keyword and subject heading search terms for four concepts: (1) medical residents; (2) gender or gender identity; (3) race, ethnicity, or foreign; and (4) bias or discrimination. The concepts were combined with Boolean AND, so all 4 concepts had to be present for a paper to be retrieved. Conference abstracts were removed. The full search strategy is provided in Multimedia Appendix 1. All search results were loaded into Covidence (Veritas Health Information) for deduplication and screening. Reference lists from all included studies were reviewed to identify additional relevant studies.

### Study Screening

Reports were screened through a multiphase process. Two researchers reviewed the first hundred papers, and the kappa coefficient was calculated to assess their agreement. We obtained a kappa of 0.72, indicating substantial agreement. Subsequently, all papers were screened based on title and abstract by 2 independent reviewers (IJ and JN), who either included or excluded each paper. A third reviewer (MR) resolved any discrepancies. During the full-text review phase, the 2 reviewers (IJ and JN) made the final decision to include or exclude each paper from the scoping review. Any discrepancies were resolved through group consensus.

### Data Extraction

Study content was extracted using Microsoft Word to create tables for organizing and managing the information from the included studies. We extracted authors’ details, the country where the studies were conducted, publication dates, sample sizes, research methods or study designs, and disciplines (Table 1). Additionally, we extracted study aims or objectives and findings. Two reviewers (IJ and JN) extracted data from all included studies, and a third reviewer (MR) double-checked the content and entries for accuracy, ensuring no data was overlooked. Any inconsistencies were resolved through group consensus. A quality appraisal of these reports was not performed, as it is not a requirement for scoping reviews [16].

**Table 1.** Characteristics of studies included in the literature review.

Studies	Country	Title	Sample (n)	Level of training	Disciplines	Study design
Zaeem et al [17]	Canada	Workplace discrimination and harassment among Alberta postgraduate medical trainees: a cross-sectional survey	195	PGY1-PGY4^a^	Anesthesiology, emergency medicine, family medicine, public health, internal medicine, dermatology, neurology, pediatrics, psychiatry, radiology, laboratory medicine specialties, and surgical specialties	Mixed methods
Kim et al [18]	United States	Evaluating the impact of gender, race, and training year on internal medicine residents’ experiences across the United States	176	PGY1-PGY4+	Internal medicine	Quantitative
Lodha et al [19]	United States	Evaluating the impact of gender and race on otolaryngology resident experiences across the United States	61	PGY1-PGY5	Otolaryngology	Quantitative
Chiraroekmongkon [20]	United States	The invisibility cloak: narrative from an Asian American woman physician-in-training	N/A^b^	N/A	Psychiatry	Personal account
Koech et al [21]	United States	Minority resident physicians’ perspectives on the role of race, ethnicity, culture, and gender in their surgical training experiences	23	PGY1-PGY4	Surgical specialties: orthopedic surgery, neurosurgery, ENT^c^, general surgery, plastic surgery, and urology	Qualitative
Hussain et al [22]	United Kingdom	The impact of race and gender-related discrimination on the psychological distress experienced by junior doctors in the UK: a qualitative secondary data analysis	14	PGY1-PGY5	General pediatrics, obstetrics and gynecology, medicine, and emergency	Qualitative
Tyler [23]	United States	CORR^d^ insights: the majority of Black orthopedic surgeons report experiencing racial microaggressions during their residency training	N/A	N/A	Orthopedic surgery	Commentary
Brooks et al [24]	United States	The majority of Black orthopedic surgeons report experiencing racial microaggressions during their residency training	310	PGY1-PGY4	Orthopedic surgery	Quantitative
Patel et al [25]	United States	Diversity, equity, and inclusion among anesthesiology trainees	135	CA^e^ 0-CA 3	Anesthesiology	Quantitative
Sandoval-Bonilla et al [26]	Mexico	Discrimination of residents during neurosurgical training in Mexico: results of a survey before SARS-CoV-2	135	PGY1-PGY5	Neurosurgery	Quantitative
Lall et al [27]	United States	Prevalence of discrimination, abuse, and harassment in emergency medicine residency training in the US	7680	PGY1-PGY4	Emergency medicine	Quantitative
Villanueva et al [28]	Australia and New Zealand	“The odds were stacked against me”: a qualitative study of underrepresented minorities in surgical training	8	PGY1-PGY5	General surgery, otolaryngology head and neck surgery, vascular surgery, plastic and reconstructive surgery	Qualitative
Zhuo et al [29]	United States	Facilitators and barriers to allyship in academic surgery: a qualitative study	15	PGY1-PGY4	Surgery	Qualitative
Mocanu et al [13]	Canada	Intersectionality of gender and visible minority status among general surgery residents in Canada	210	PGY1-PGY5	General surgery	Quantitative
Mgbako [30]	United States	Bonds in every color	N/A	PGY1-PGY4	Internal medicine	Personal account
Yuce et al [31]	United States	National evaluation of ethnic or racial discrimination in US surgical residency programs	5679	PGY1-PGY4	Surgery	Quantitative
Pearce et al [32]	Australia and New Zealand	Gender effects in anesthesia training in Australia and New Zealand	356	PGY1- provisional fellowship	Anesthesiology	Quantitative
Hu et al [33]	United States	Discrimination, abuse, harassment, and burnout in surgical residency training	7409	PGY1-PGY5	Surgery	Quantitative
Osseo-Asare et al [34]	United States	Minority resident physicians’ views on the role of race or ethnicity in their training experiences in the workplace	27	PGY1-PGY4	Anesthesiology, emergency medicine, family medicine, internal medicine, neurology, obstetrics and gynecology, pediatrics, psychiatry, radiology, surgery, and urology	Qualitative
Fitzgerald et al [35]	United States	Screening for harassment, abuse, and discrimination among surgery residents: an EAST^f^ multicenter trial	76	PGY1-PGY5	Surgery	Quantitative
Bhatt [14]	United States	The little brown woman: gender discrimination in American medicine	108	PGY1-PGY4	Dermatology, family medicine, internal medicine, neurology, pathology, pediatrics, physical medicine and rehabilitation, psychiatry, radiology, and surgical specialties	Qualitative
Fnais et al [36]	Saudi Arabia	Prevalence of harassment and discrimination among residents in three training hospitals in Saudi Arabia	213	PGY1-PGY5	All specialties	Quantitative
Crutcher et al [37]	Canada	Family medicine graduates’ perceptions of intimidation, harassment, and discrimination during residency training	242	PGY1- PGY2	Family medicine	Mixed methods
London et al [38]	South Africa	A survey of trainee specialists’ experiences at the University of Cape Town (UCT): impacts of race and gender	91	PGY1-PGY5	Medicine, pediatrics, and psychiatry	Quantitative
vanInevald et al [39]	Canada	Discrimination and abuse in internal medicine residency	543	PGY1-PGY4	Internal medicine	Quantitative
McNamara et al [40]	United States	The extent and effects of abuse and harassment of emergency medicine residents.	1774	PGY1-PGY5	Emergency medicine	Mixed methods

a PGY: postgraduate year.

b N/A: not applicable.

c ENT: ear, nose, and throat.

d CORR: Clinical Orthopaedics and Related Research.

e CA: clinical anesthesiology.

f EAST: Eastern Association for the Surgery of Trauma.

### Synthesis of Results

Data were analyzed using conventional content analysis, a suitable approach given the lack of predefined categories. Content analysis allowed us to identify the main categories, relationships between them, and gaps in the literature. One team member (IJ) created data abstraction tables before data abstraction began. These abstraction forms included citation details, such as author, year of publication, title, and country of study, as well as study characteristics, such as study design, research objective, and sample size. Two team members (IJ and JN) coded the data and organized it into 4 themes. Any discrepancies at this stage were resolved through consensus.

### Ethical Considerations

This is a scoping review that synthesizes published papers; therefore, no human ethics approval was required.

## Results

### Description of Included Studies

**Figure 1.**
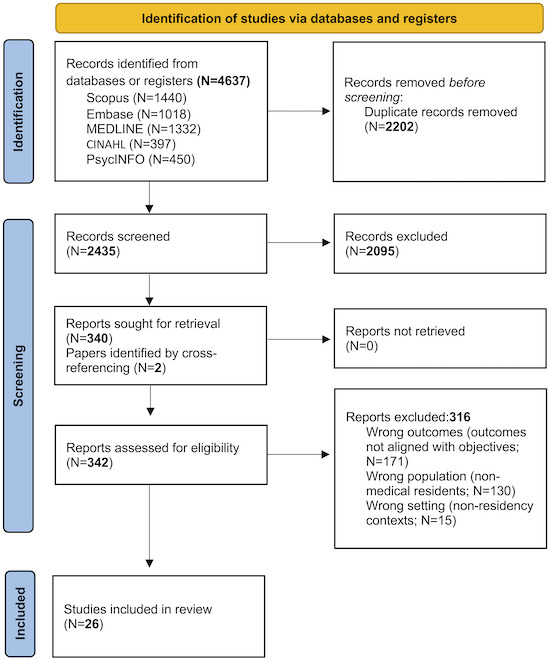
PRISMA-ScR (Preferred Reporting Items for Systematic Reviews and Meta-Analyses Extension for Scoping Reviews) flow diagram for the papers included in this study.

The initial search identified 4637 potential studies, of which 2435 remained for title and abstract screening after duplicates were removed. A total of 340 papers were selected for full-text review, and ultimately, 24 papers met the inclusion criteria. Reviewing the reference lists of the included papers uncovered 2 additional relevant reports, bringing the total to 26 studies included in the scoping review (Figure 1).

An overview of the characteristics of the included studies is provided in Table 1. These papers were published between 1995 and 2024. Most studies (n=16) were conducted in the United States, followed by Canada (n=4), Australia and New Zealand (n=2), and 1 study each from Saudi Arabia, Mexico, South Africa, and the United Kingdom. Fourteen papers used a quantitative study design, followed by 6 qualitative studies, 3 mixed-methods studies, 2 personal accounts, and 1 commentary. Of the 23 research studies, sample sizes ranged from 8 to 7409. Residents came from a variety of specialties, including mixed specialties (n=8), surgery (n=5), internal medicine (n=3), anesthesiology (n=2), emergency medicine (n=2), orthopedic surgery (n=2), otolaryngology (n=1), family medicine (n=1), neurosurgery (n=1), and psychiatry (n=1).

Our data analysis revealed four themes: (1) forms of discrimination faced by VMs, (2) sources of discrimination, (3) ramifications of discrimination, and (4) ways to reduce gender and racial discrimination (Table 2).

**Table 2.** Key findings of included studies.

Studies	Objectives	Key findings	Themes reported
Zaeem et al [17]	The goal of this study was to characterize the demographics and experiences of discrimination faced by residents to improve their learning environment.	More women than men have frequently experienced a form of discrimination, harassment, or mistreatment. No differences in the frequency of discrimination experienced were found between residents who are Black, Indigenous, or People of Color (BIPOC) and White residents; however, BIPOC residents reported hearing more racial slurs, either about them or in general. They also provided examples of how attendings often negatively commented on their work, leading to differential treatment of women and BIPOC residents compared to other groups.	Theme 1: the forms of discrimination faced by VM and Theme 2: sources of discrimination
Kim et al [18]	This study aimed to characterize how resident well-being and discrimination experienced by residents in internal medicine were impacted by their race, gender, and training year.	More women than men reported experiencing differential treatment from faculty and receiving fewer opportunities due to their gender. Residents of ethnic backgrounds described being treated differently by faculty, with mostly Black residents reporting facing discrimination and receiving fewer opportunities due to their race. The majority of Black residents and female residents reported being misidentified for another role, which contributed to feelings of burnout reported by female residents. Finally, the authors suggested that residency programs provide explicit and implicit bias training to residents and staff to reduce discrimination.	Theme 1: the forms of discrimination faced by VM, Theme 3: ramifications of discrimination, and Theme 4: ways for individuals and institutions to reduce discrimination
Lodha et al [19]	The purpose of this study was to determine if otolaryngology residents’ experiences with discrimination and burnout varied by gender and race.	Many women and underrepresented minorities (URMs) reported experiencing misidentification as a nonphysician and perceived receiving fewer opportunities in research and training compared to men. Twenty-seven percent of women described experiencing burnout, and 40.5% of women felt less confident in their ability to independently practice. The authors recommended raising awareness among residents regarding these experiences and training them on how they can support others who may face discrimination.	Theme 1: the forms of discrimination faced by VM, Theme 3: ramifications of discrimination, and Theme 4: ways for individuals and institutions to reduce discrimination
Chiraroekmongkon [20]	This narrative captures the experiences of an Asian American woman with discrimination during residency.	The author described facing gender and racial discrimination from patients, including verbal abuse and other inappropriate comments. She also faced discrimination from colleagues through her ideas being credited to someone else and being confused with other Asian colleagues. Discrimination also exists institutionally, as Asian Americans are not considered underrepresented in medicine since they make up a representative portion in health care. However, this does not remove the discrimination they continue to face. Experiencing discrimination can result in internalization of these negative messages and silencing of one’s identity.	Theme 1: the forms of discrimination faced by VM, Theme 2: sources of discrimination, and Theme 3: ramifications of discrimination
Koech et al [21]	The goal of this study was to determine how surgical resident training experiences were impacted by race, gender, and cultural background.	The majority of URMs described being treated differently, having difficulties fitting in, and experiencing microaggressions from various sources. They were also more likely to face greater criticism during training compared to their non-URM coresidents. Many residents combated these issues by attempting to assimilate, isolate, or seek assistance from outside sources. Participants suggested educational interventions for reducing discrimination. This included formal sessions, such as URM sharing their experiences, and informal sessions, where a senior resident or attending addresses an instance of discrimination as it occurs on the ward.	Theme 1: the forms of discrimination faced by VM, Theme 3: ramifications of discrimination, and Theme 4: ways for individuals and institutions to reduce discrimination
Hussain et al [22]	The objective of this qualitative study was to examine the experiences of racial and gender discrimination perceived by residents and its effects on their psychological distress (PD).	Many participants experienced different forms of discrimination, including microaggressions and explicit racism. Black, Asian, and minority ethnic (BAME) women experienced more discrimination than White women and BAME men. Patients were a common source of discrimination, with some requesting to see White physicians instead. Senior physicians and midwives were also described as perpetrators of discrimination. Participants reported feeling higher levels of PD, which was positively correlated with experiencing discrimination. Participants proposed improving the reporting system, providing educational sessions regarding discrimination, and making residents aware of the policy that allows them to refuse noncritical care to prejudiced patients.	Theme 1: the forms of discrimination faced by VM, Theme 2: sources of discrimination, Theme 3: ramifications of discrimination, and Theme 4: ways for individuals and institutions to reduce discrimination
Tyler [23]	The author of this commentary aimed to provide suggestions for improving the recruitment and retention of women and URMs into orthopedic residency.	One major suggestion described raising awareness among program directors and faculty by providing training on microaggressions and giving nonexclusionary feedback. Other suggestions included residents being provided with ways to report without fear of retribution, providing early support to Black women or those who face intersectional experiences, and acknowledging that these incidents occur.	Theme 1: the forms of discrimination faced by VM and Theme 4: ways for individuals and institutions to reduce discrimination
Brooks et al [24]	This study aimed to determine the degree to which Black residents experienced discrimination, the different types of microaggressions they faced, and whether there were differences in the discrimination faced by Black women and men.	The majority of participants reported experiencing either a lot of discrimination (34%) or some discrimination (44%) at their workplace from patients and faculty. Around 87% of participants described being misidentified as a nonphysician, and 81% of participants reported being misidentified for a nonmedical position. Fifty percent of participants also received devaluing feedback, with 87% reporting it to be based on race. Additionally, when comparing for gender, more Black women reported experiencing discrimination through microaggressions and receiving feedback than Black men. These forms of feedback often caused feelings of “othering.” The authors listed some common forms of negative feedback to avoid and also recommended that leadership provide safe spaces for residents to report.	Theme 1: the forms of discrimination faced by VM, Theme 2: sources of discrimination, Theme 3: ramifications of discrimination, and Theme 4: ways for individuals and institutions to reduce discrimination
Patel et al [25]	This study aimed to determine residents’ intentions in choosing anesthesiology, evaluate the extent of discrimination experienced by residents, and provide a foundation for implementing change in the future.	The majority of respondents identified mentors and role models as the most significant reason for choosing anesthesiology. More women than men also reported that women and diverse faculty were another important factor in their decision. More non-White residents reported facing racial discrimination during the residency application process and during residency training. Around 35.1% of women also faced gender discrimination during their training. Consequently, increasing the number of underrepresented minorities and women in leadership positions may assist in reducing discrimination.	Theme 4: ways for individuals and institutions to reduce discrimination
Sandoval-Bonilla et al [26]	The purpose of this study was to describe the discrimination experienced by neurosurgical residents.	Twenty-seven percent of participants experienced a type of discrimination, with the majority of experiences being due to place of origin, gender, or physical appearance. More women than men reported experiencing gender discrimination, and more foreign residents than Mexican residents reported facing discrimination due to place of origin. Participants who did not experience discrimination were more likely to report being satisfied with their education.	Theme 3: ramifications of discrimination
Lall et al [27]	This study aimed to evaluate the prevalence, forms, and causes of discrimination, abuse, and harassment experienced by residents in emergency medicine. They also sought to determine the association between these forms of mistreatment and suicidality.	Around 45.1% of participants reported experiencing 1 form of mistreatment (discrimination, abuse, or harassment) during the recent year, and many identified patients and patients’ families as the main cause. More women than men described facing gender discrimination and sexual harassment, while residents of ethnic backgrounds described facing more racial discrimination than their White peers. Suicidality was equally prevalent among male and female residents, although adjusting for mistreatment significantly decreased suicidality among female residents. Educating residents and staff through cultural competency training may help with creating an inclusive environment for residents.	Theme 1: the forms of discrimination faced by VM, Theme 2: sources of discrimination, Theme 3: ramifications of discrimination, and Theme 4: ways for individuals and institutions to reduce discrimination
Villanueva et al [28]	The purpose of this study was to determine the barriers and examine the experiences of URM residents in choosing and completing the Surgical Education and Training (SET) program.	Women residents reported being discouraged from applying for surgery due to the possibility of raising a family in the future. International medical graduates (IMGs) reported experiencing racial discrimination, especially if they had an accent. Women also experienced sexual harassment and felt their opinions were undervalued by their male peers. As a result, many women reported feeling lonely or alienated. Recruiting more underrepresented minorities in leadership positions would provide additional role models for trainees.	Theme 1: the forms of discrimination faced by VM, Theme 3: ramifications of discrimination, and Theme 4: ways for individuals and institutions to reduce discrimination
Zhuo et al [29]	The goal of this qualitative study was to describe the covert types of biases that residents experienced and to explore the different approaches that residents took when addressing them.	Most participants reported experiencing biases based on their gender and race. These included patients requesting a physician of a different race and individuals in senior positions making subtle, derogatory comments about residents. Residents struggled with recognizing discrimination when it occurred, which affected when and how they responded. They were less likely to address bias if it was caused by someone in a position of power and if they believed that responding would take up a lot of energy. Participants mentioned that representation and administrative support influenced whether they responded to incidents or not.	Theme 1: the forms of discrimination faced by VM, Theme 2: sources of discrimination, Theme 3: ramifications of discrimination, and Theme 4: ways for individuals and institutions to reduce discrimination
Mocanu et al [13]	The objective of this study was to explore the effects of visible minority (VM) status and gender on the experiences of surgical residents.	Around 81.2% of women and 14.3% of those of VM status described being dismissed for their medical expertise due to their gender and race, respectively. Women of VM status and women in general are perceived to receive fewer opportunities due to their gender. Women of VM status were also more likely to report challenges with fitting in, forming good relationships with staff, and being appreciated. Institutions and individuals must be aware that these biases exist, and increasing the number of mentors and role models may assist with achieving a culture of equality.	Theme 1: the forms of discrimination faced by VM, Theme 3: ramifications of discrimination, and Theme 4: ways for individuals and institutions to reduce discrimination
Mgbako [30]	This commentary recounts a meeting where physicians and residents of color shared their experiences of discrimination.	Residents shared times when patients questioned their abilities due to their race, whether through comments on their accent or asking to be seen by another physician. During these incidents, residents reported suppressing their feelings and experiencing shame for not being able to defend themselves.	Theme 1: the forms of discrimination faced by VM and Theme 3: ramifications of discrimination
Yuce et al [31]	This study aimed to discover the prevalence, sources, and factors of racial discrimination and its impact on residents’ well-being.	Non-White participants reported facing more discrimination due to race or ethnicity than White participants, with a larger portion (70.7%) of Black residents reporting racial discrimination than any other racial group. Most Black residents described being confused with other Black residents, experiencing role misidentification, being evaluated differently, and experiencing racial slurs. Patients were the most reported source of racist comments, while nurses and staff were most likely to confuse residents of the same race. Burnout and suicidal thoughts were more likely to be reported by residents who experienced discrimination. The authors recommended training residents and faculty to recognize and intervene during incidents and to encourage residents to report their experiences.	Theme 1: the forms of discrimination faced by VM, Theme 2: sources of discrimination, Theme 3: ramifications of discrimination, and Theme 4: ways for individuals and institutions to reduce discrimination
Pearce et al [32]	The objective of this survey was to evaluate the differences between male and female anesthesiology residents regarding the number of procedures performed, their confidence in their abilities, and whether they face gender and ethnic discrimination.	Male residents reported conducting procedures >10 times as frequently compared to female residents. They were also more likely to rate their abilities higher than their training level and to report greater competence with procedures to receive more opportunities. More female residents reported experiencing gender discrimination than male residents, which was more pronounced in female residents of VM status. Consequently, the authors suggested making faculty aware of these implicit biases and ensuring that women residents receive the same opportunities as male residents.	Theme 1: the forms of discrimination faced by VM and Theme 4: ways for individuals and institutions to reduce discrimination
Hu et al [33]	This survey aimed to identify the frequency and prevalence of experiences of mistreatment, burnout, suicidal thoughts, and their relationship with each other.	Women reported experiencing more gender discrimination and sexual harassment than male residents. Both gender and racial discrimination were reported to be caused mostly by patients. Around 38.5% of residents experienced burnout weekly, while 4.5% reported experiencing suicidal thoughts. Women who experienced mistreatment were more likely to experience burnout.	Theme 1: the forms of discrimination faced by VM, Theme 2: sources of discrimination, and Theme 3: ramifications of discrimination
Osseo-Asare et al [34]	The objective of this study was to identify and describe how race or ethnicity impacts resident experiences.	Residents reported experiencing microaggressions, including being asked questions regarding their ethnicity, being misidentified for another role by patients and staff, experiencing exoticization or being mistaken for another resident of color, and experiencing explicit racism. Other themes included being burdened with tasks related to diversity and struggling to balance professional and personal identities at work. These experiences could be improved upon by providing residents with forums to discuss these issues and by hiring more VMs to provide mentorship.	Theme 1: the forms of discrimination faced by VM, Theme 2: sources of discrimination, Theme 3: ramifications of discrimination, and Theme 4: ways for individuals and institutions to reduce discrimination
Fitzgerald et al [35]	This study sought to characterize the prevalence of harassment, abuse, and discrimination experienced by surgical residents using the HITS (Hurt, Insulted, Threatened with harm, or Screamed at) tool.	About 77.6% of participants experienced abuse and harassment, mostly receiving insults or being screamed at. The majority of residents reported experiencing sexual harassment, gender discrimination, and racial discrimination. Patients and supervising physicians were the most reported sources of discrimination. More women experienced gender discrimination, which has negatively impacted their environment and performance. Researchers also found that residents who reported gender discrimination were also more likely to report experiencing racial discrimination. They recommended training residents on how to report harassment and making them aware of accessible resources.	Theme 1: the forms of discrimination faced by VM, Theme 2: sources of discrimination, Theme 3: ramifications of discrimination, and Theme 4: ways for individuals and institutions to reduce discrimination
Bhatt [14]	The objective of this study was to explore the experiences of gender discrimination faced by residents of Indian origin and examine the interaction of gender with race in their careers.	Many participants described facing various forms of gender discrimination, starting at the residency application process, where women were asked about their plans for having children. Women were also more likely to be guided toward less labor-intensive specialties, such as pediatrics and family medicine. Race and gender together elevated their risk of facing discrimination, which was further exacerbated if residents were trained in a foreign country. Being a woman and of VM status also affected their chances of reaching leadership positions.	Theme 1: the forms of discrimination faced by VM and Theme 3: ramifications of discrimination
Fnais et al [36]	The purpose of this study was to determine how prevalent harassment and discrimination are during residency training.	Most participants experienced negative verbal comments during residency training, with consultants being the primary source. Rates of gender discrimination were reported equally by female and male residents; however, more female residents reported experiencing sexual harassment. Many participants reported wanting a different career. The authors recommended providing a confidential reporting system to allow residents to report their experiences.	Theme 1: the forms of discrimination faced by VM, Theme 2: sources of discrimination, Theme 3: ramifications of discrimination, and Theme 4: ways for individuals and institutions to reduce discrimination
Crutcher et al [37]	This study aimed to explore how intimidation, harassment, and discrimination (IHD) affected the experiences of family medicine residents.	About 44.7% of respondents experienced a form of IHD as a resident, with 94.3% experiencing negative verbal comments. The form of gender discrimination varied based on gender, with more female residents perceiving that they received fewer opportunities, while male residents perceived receiving more work as punishment. Additionally, IMGs were perceived to experience more discrimination based on their ethnicity and language than Canadian medical graduates. Observing resident well-being may be one way to measure experiences of IHD.	Theme 2: sources of discrimination, Theme 3: ramifications of discrimination, and Theme 4: ways for individuals and institutions to reduce discrimination
London et al [38]	The goal of this study was to identify the demographics of residents enrolled from 1999 to 2006 and to determine factors that impacted the recruitment and retention of Black female residents.	The number of VMs and women increased from 1999 to 2006; however, some participants reported knowing others who chose to go to other schools due to reports of racial discrimination. More Black residents than White residents reported feeling unwelcome. Fourteen participants experienced discrimination, but only half reported racial discrimination.	Theme 1: the forms of discrimination faced by VM and Theme 3: ramifications of discrimination
vanInevald et al [39]	This study aimed to explore the experiences of gender and racial discrimination and homophobia among residents in internal medicine.	Female residents revealed higher rates of gender discrimination, with patients (88%) and attendings (70%) being indicated as major sources. More female residents also reported experiencing sexual harassment, with 56% reporting patients and 35% reporting attendings as the main problem. Patients continued to be a major source for racial discrimination (67%), followed by other health care staff (52%), peers (50%), and attendings (49%). The authors suggested training residents on how to deal with patients causing discrimination.	Theme 1: the forms of discrimination faced by VM, Theme 2: sources of discrimination, and Theme 4: ways for individuals and institutions to reduce discrimination
McNamara et al [40]	The objective of this study was to assess the degree to which harassment and racial discrimination are experienced by residents in emergency medicine and how these experiences impact their well-being.	Ninety-eight percent of participants reported experiencing abuse or harassment, with women reporting higher rates of sexual harassment and differential treatment due to gender. Non-Whites reported experiencing racial discrimination more than White residents. Patients were the major cause of most discriminatory experiences. Encounters of discrimination left participants questioning their choice of becoming a physician and choosing emergency medicine. The researchers suggested providing educational sessions for residents, establishing reporting systems, and providing support and counseling.	Theme 1: the forms of discrimination faced by VM, Theme 2: sources of discrimination, Theme 3: ramifications of discrimination, and Theme 4: ways for individuals and institutions to reduce discrimination

### Theme 1: Forms of Discrimination Faced by VMs

Twenty-three (88%) studies highlighted the different ways discrimination has manifested against VMs [13,14,17-24,27-36,38-40]. Eighteen papers described instances of microaggressions that were experienced by women and VM residents [13,14,17-22,24,28-32,34,35,38,40]. Participants in 11 studies reported experiencing misidentification of their role due to their gender and/or race [13,17-20,22,24,29,31,32,34]. Most residents who were assumed to be a nonphysician or a non–health care worker were women [13,17-20,22,29,32], of VM status [18,19,24,31,34], or both [18,24,32]. Women were more likely to be assumed to be a nurse or secretary [20,22,29], while VMs were more likely to be assumed to be custodial staff [24,29]. Moreover, residents experienced misidentification even after introducing themselves and wearing a white coat and stethoscope [34]. However, residents were aware that misidentification likely occurs due to stereotypes [29]. In addition to misidentification of role, VMs also reported being mistaken for another resident of minority status [20,31,34] and having patients ask to see a physician of another race [22,29,30].

VM residents in 10 papers also reported experiencing verbal microaggressions [17,20-22,24,29,31,34,35,40], including hearing racial slurs [17,31,40], receiving questions about their ethnicity [22,34], and astonished comments on their English proficiency [17,22], such as “Well, at least you speak good English!” [17]. The majority of these microaggressions originated from patients; however, attendings and other health care workers were a close second [21,24,29,31,34,40]. VMs were also discriminated against for their accents, often being treated with less respect as a result [14,28,30].

Another frequently reported form of microaggression across 5 studies was that VM residents were assessed using different standards than their non-VM coresidents [14,17,21,24,31]. This disparity manifested as harsher evaluations [17,21] and more critical feedback on performance [24].

Most gender-based microaggressions occurred against women. For example, women residents in 3 papers were more likely to report facing discrimination due to pregnancy and childcare than men residents [14,27,33]. These included women being asked about their family plans during residency interviews, being treated differently while pregnant during residency, and facing a lower chance of receiving a promotion [14]. Additionally, many faculties discouraged women from entering competitive disciplines, such as surgery, due to these specialties being considered labor-intensive and incompatible with family responsibilities [14,22,28]. Women were also more likely to face discrimination from other women, attending physicians, and nurses [14,22].

Furthermore, many studies recognized that microaggressions were compounded and worse for women of VM status [13,14,18,22,24,31,32,35]; however, only 3 studies investigated resident experiences with an intersectional lens [13,14,22]. One study found that VM women were less likely to feel that they had a good relationship with their staff [13]. Another study found that Black, Asian, and minority ethnic women often felt sidelined by men and found it overwhelming to respond to every instance of discrimination [22].

Another form of discrimination that was reported less frequently but consistently over the years is sexual harassment, which was primarily reported by women in 10 papers [20,22,27-29,33,35,36,39,40]. Inappropriate sexual comments [20,22,28,29,35,36] and unwanted sexual jokes [22,35,40] were the most commonly described, followed by sexual advances [36,40]. Other instances of sexual harassment include unwanted attention and disrespectful body language [36].

### Theme 2: Sources of Discrimination

Fourteen (53%) papers explored who contributed to the discrimination experienced by VMs and women [17,20,22,24,27,29,31,33-37,39,40]. Our literature review found that VMs experienced discrimination from a multitude of perpetrators. These include, but are not limited to, patients, families, attendings, peers, nurses, and other health care professionals [17,20,22,24,27,29,31,33-37,39,40].

Participants in 12 studies reported patients and patients’ families as either the main or the second most common perpetrators of discrimination [20,22,24,27,29,31,33-36,39,40]. Of those who reported patients as the main source, these experiences of discrimination were both racial [20,22,24,27,29,31,33,34,39,40] and gender-based [27,29,33,39]. Patients were also the most common cause of sexual harassment against women residents, followed by staff, including attending physicians and nurses [20,27,29,33,39,40].

Eight studies reported that attending physicians were another major contributor to the racial [17,24,29,33-35,39] and gender [33,35,36,39] discrimination experienced by VM residents. Attending physicians were also found to be a major cause of sexual harassment [33,35,39], with 1 paper reporting them to be the main perpetrators [35]. They highlighted the negative impact this can have on residents, as these attendings are often their mentors who serve as guides for their education and, therefore, can influence resident learning environments [35]. Eight papers described how VM residents often experienced discrimination from nursing staff and other health care workers [17,22,27,31,33,34,37,39]. Only 4 papers mentioned peers and colleagues being a cause of discrimination [17,33,34,39].

### Theme 3: Ramifications of Discrimination

Twenty-one (80%) papers found that participants experienced the ramifications of discrimination in a variety of ways, with many papers exploring the effects of discrimination and how they manifest [13,14,18-22,24,26-31,33-38,40]. A major consequence of discrimination was residents being reluctant to report their experiences [13,14,22,28,29,34,35,40]. Many participants described being afraid that reporting these instances could have repercussions for their professional career [22,29,34,35,40], especially for those who experienced discrimination from senior colleagues and leadership [22,29,34]. Participants also avoided reporting incidents because they did not have the time and energy to do so [14,22,29,34]. Some residents mentioned being worried that their efforts to report would be wasted, as their experiences would just be swept under the rug [13,34,40]. Residents also felt that their experiences were not significant enough to report [22,35,40]. One paper mentioned residents being worried that their report would be perceived as playing the “race card” [34].

Five papers found that learners experienced burnout due to discrimination [18,19,29,31,33], with 1 paper reporting that learners felt more fatigued and exhausted on top of their current program workload and expectations [29]. Four studies spoke about the feelings of loneliness [21,24,28,34], with extreme cases of learners experiencing feelings of being “othered” [21,24]. Discrimination also caused some residents to have suicidal thoughts [23,27,33], with studies finding an association between mistreatment exposures and the number of suicidal thoughts [27,33]. Learners also reported that discrimination caused them to feel the need to suppress their feelings, resulting in residents ignoring the incident altogether [29,30]. A few residents mentioned internalizing messages of discrimination [20,22], making them feel less competent in their clinical abilities [22] or feeling the need to hide their culture [20]. Residents who experienced discrimination reported poorer learning environments [35,38]. Similarly, in 1 study, residents who did not experience discrimination were more likely to rate their academic satisfaction [26] highly. As a result, many women and/or VM residents mentioned contemplating withdrawing from their residency program or quitting entirely [13,31,35,36,40].

### Theme 4: Ways for Individuals and Institutions to Reduce Gender and Racial Discrimination

Nineteen (73%) studies discussed and provided potential solutions for reducing gender and racial discrimination experienced by residents [13,18,19,21-25,27-29,31,32,34-37,39,40]. These recommendations ranged from educational interventions that could support residents to changes that could be implemented at the institutional level.

Many papers recommended various curricular and training changes to reduce discrimination [18,19,21-23,27,29,31,32,35,39,40]. These included providing training on what explicit and implicit biases are and how to recognize them [18,19,21-23,27-29,32,35,40], as well as incorporating training on how to respond and intervene during instances of discrimination [18,19,22,27,29,31,39]. In addition to training residents, some papers suggested providing faculty and other health care professionals with these educational opportunities as well [18,21-23,29,32,35].

Several studies also proposed increasing the number of women and underrepresented minorities in faculty and leadership positions as a way for institutions to foster an inclusive environment [13,21,25,28,34]. Many participants believed that hiring more minorities into positions of power would allow more residents access to mentors who could provide the support and resources they need while facing challenges in residency [13,21,25,28,34].

Some papers suggested that institutions should set policies to improve working conditions for women and VMs. These included encouraging residents to report incidents when they occur [22-24,31,35,36,40] and keeping them safe from retribution [23,24,36,40] by using a confidential reporting system [36]. Another suggestion was to implement policies that required residents to refer offending patients to other physicians, thereby allowing residents to work in a safe environment while ensuring that patients received the care they needed [22].

## Discussion

### Principal Findings

The purpose of this scoping review was to explore and highlight resident experiences of gender and racial discrimination to raise awareness of the impact of these experiences on residency training programs. The findings revealed that residents predominantly reported experiencing microaggressions, misidentification, and sexual harassment from multiple sources, including attendings and other health care workers. These experiences frequently had negative repercussions, including adverse effects on residents’ feelings of competency.

### Educational Impact of Microaggressions

Discrimination experienced by VMs is an ongoing issue that has plagued the health care system for a very long time. Microaggressions are becoming an increasingly common form of discrimination experienced by residents and physicians of VM status [2,34]. Several studies allude to discrimination toward medical trainees as part of their training due to factors such as hierarchy [41,42]. A recent study by Gianakos et al [43] found that the majority of incidents reported by residents in the study were perpetrated by supervising attendings and their senior coresidents. Similarly, our analysis found that attendings were the most common source of discrimination, second only to patient interactions. Microaggressions from attendings and other senior colleagues can have serious and lasting consequences for residents, particularly with respect to career trajectory. The impacts of these microaggressions on future careers can start well before residency; for example, many women reported being discouraged by faculty from entering competitive specialties in residency, such as surgery, preventing these disciplines from becoming diverse [14,22,28]. Furthermore, microaggressions that occur during residency also impact resident careers. For instance, our findings indicate that residents from marginalized communities are commonly subjected to higher standards and are more likely to receive harsher evaluations than their non-VM peers [21]. Such discriminatory feedback can negatively affect residents’ progress during training and may also discourage them from pursuing careers in academia [44]. Additionally, several papers reported that residents expressed concern about receiving fewer opportunities based on their gender and/or race [13,18,19,22,35,37]. Residents may also lack sponsorship and general support from attending physicians, which can limit access to career-advancing opportunities, including employment at prestigious academic institutions [44]. Consequently, fewer women and VMs attain leadership positions, resulting in reduced availability of mentorship and advocacy for marginalized residents [13,21].

Similar patterns have been observed among faculty physicians. Faculty identifying as VMs report feeling pressured to meet higher performance standards than their non-VM colleagues to maintain their positions or advance professionally [45]. This differential treatment may further contribute to the underrepresentation of VMs in senior academic and leadership roles [45].

### Intersectionality

The compounded effects of discrimination faced by residents with intersectional identities were acknowledged in many papers; however, they were not explored in depth. Learning about the unique experiences of residents who face discrimination due to both their gender and race is essential for developing solutions, since these issues cannot be resolved by addressing gender or racial discrimination alone [13]. The intersectional experiences of physicians described in the literature are similar to those reported by residents. A study that interviewed Black female physicians found that many of them felt isolated from their colleagues and patients, similar to how residents were less likely to feel connected to staff [13,46]. Physicians also described feeling burdened with high expectations due to their race and gender, while many did not expect them to succeed [46]. More qualitative studies on the intersectional experiences of residents are necessary to reveal how they may differ from or relate to physician experiences.

### Strategies for Reducing Discrimination and Microaggressions

Many papers within this scoping review proposed strategies for reducing gender and racial discrimination against residents. Although many papers recommended incorporating training on discrimination and intervening in situations of microaggressions, none of the papers mentioned implementing these suggestions within their institutions or evaluating the impact of these solutions on resident experiences. It is important to highlight that this is a notable gap in the literature, as there are many studies describing discrimination and possible solutions for reducing these experiences, but very few studies that assess the efficacy of these recommendations. A study conducted by Mullett et al [47] focusing on equity, diversity, and inclusion curriculum development for pediatric residents revealed that when residents were exposed to training sessions on microaggressions, their knowledge and awareness about microaggressions in a clinical setting increased. Additionally, the residents were able to propose effective strategies to deal with microaggressions.

In addition to providing educational opportunities for residents, several papers suggested training faculty and leadership about discrimination [18,21-23,29,32,35] and how to respond and intervene during these situations [22,27,31]. Including staff and faculty in these educational sessions could motivate witnesses to intervene when an incident related to discrimination occurs [22,27,31]. Similar to the literature on residents, there is limited research evaluating the impact of such interventions on faculty. Gleeson et al [48] described the development and outcomes of a diversity, equity, and anti-racism curriculum implemented at their medical center. The curriculum aimed to raise awareness and equip physicians and trainees with skills to recognize racism through didactic sessions and interactive workshops. Although participants reported increased knowledge and rated the sessions as impactful, survey data collected longitudinally showed no change in the institutional discrimination experienced by participants or by individuals they knew.

Another strategy for decreasing discrimination was to increase the number of women and VMs in leadership positions, particularly for expanding the number of mentors and role models available for residents [13,21,25,28,34]. Mentorship is considered to be one of the most significant aspects of residency that can greatly impact a resident’s experience, and studies show that women of VM status are the least likely to have a mentor during residency [13]. In addition to mentorship, VMs recruited into positions of leadership would be more likely to be advocates for residents who identify as women and VMs [21].

### Implications

Findings from this scoping review generated new perspectives and knowledge to better understand how race and gender can combine to impact resident experiences. This scoping review illuminated areas of postgraduate medical education that need change and highlighted additional actions required to enhance resident experiences. Understanding how gender and racial discrimination intersect and manifest at the postgraduate level can aid programs in developing a tailored response to these challenges. Improving resident experiences and supporting them in addressing these issues can positively influence their interactions with colleagues and patients, leading to improved patient care. Additionally, raising awareness of this topic can educate residents, physicians, and other staff, fostering better treatment of colleagues based on their gender and race. The results of this scoping review will contribute to postgraduate medical programs evaluating their curricula and refining or incorporating training on discrimination within existing programs.

### Strengths and Limitations

This scoping review was based on a rigorous search and methodological process. The search strategy included many synonyms and was adapted for 5 databases. We followed the PRISMA (Preferred Reporting Items for Systematic Reviews and Meta-Analyses) reporting checklist, which added to the rigor of our scoping review. However, some limitations should be acknowledged. We did not impose date restrictions because we aimed to examine how residents’ experiences with discrimination may have changed over time. However, it is important to note that the composition of the resident workforce has evolved substantially during this period, particularly with an increase in the proportion of women in medical training. Although insufficient historical data precluded direct comparisons across earlier periods, our findings suggest that residents’ experiences of discrimination have remained largely similar over time. Some studies may have been overlooked due to our search being limited to papers in English. Additionally, although we examined studies from different countries, our findings may not be generalizable to all residency programs due to differing societal contexts, varying norms, and possibly different training structures.

### Conclusion

Findings from this scoping review describe the different ways women and VM residents often face discrimination, who perpetrates it, the consequences it may have on learners, and steps that individuals and institutions can take to reduce discrimination. Even though residents have been reporting experiences of gender and racial discrimination for many years, discrimination continues to be a persistent and ongoing issue in many residency programs. Learning about and raising awareness of resident experiences can assist institutions and residency programs in developing tailored solutions, especially for those who face intersectional discrimination.

## Supplementary material

10.2196/87524 Multimedia Appendix 1 Search strategy.

10.2196/87524 Checklist 1 PRISMA-ScR (Preferred Reporting Items for Systematic Reviews and Meta-Analyses Extension for Scoping Reviews) checklist.

## Abbreviations

PRISMA: Preferred Reporting Items for Systematic Reviews and Meta-Analyses
PRISMA-ScR: Preferred Reporting Items for Systematic Reviews and Meta-Analyses Extension for Scoping Reviews
VM: visible minority
