# Supplementary material for: Gender and Racial Discrimination During Residency Training: Scoping Review
Source: JMIR Med Educ. 2026 Apr 2;12:e87524. doi: 10.2196/87524 (PMC13045779; doi:10.2196/87524)
Supplement: Multimedia Appendix 1 [file mededu-v12-e87524-s001.docx]

**Ovid MEDLINE(R) ALL <1946 to May 01, 2025>**

Date searched: May 2, 2025

Results: 1332

1 exp *Education, Medical, Graduate/ or *Faculty, medical/ or ("residen* education" or "residen* training" or "fellowship training" or (residen* adj3 (intern or interns or internship*)) or PGE or PGME or "clinical faculty" or "clinical instructor*" or "clinical preceptor*" or "medical faculty" or "surgical faculty" or ((resident or residents or residency or residencies or intern or interns or internship* or fellow* or graduate or postgraduate or post-graduate or "academic faculty" or "academic staff" or "teaching staff" or "research faculty" or "diverse faculty" or "minority faculty" or "junior faculty" or "faculty members" or "female faculty" or "male faculty" or professor* or tenure-track or tenured or "department* chair*") and (medical or medicine or surgical or surgery or anesthesiolog* or anaesthesiolog* or cardiology or dermatolog* or endocrinology or gastroenterolog* or "general practice" or gerontolog* or gynaecolog* or gynecolog* or haematolog* or hematolog* or hepatolog* or immunolog* or neonatolog* or nephrolog* or neurolog* or neurosurg* or obstetric* or ophthalmolog* or oncolog* or orthopedic* or orthopaedic* or otolaryngolog* or otorhinolaryngolog* or paediatric* or pediatric* or pathology or podiatry or physiatry or psychiatr* or pulmonolog* or radiolog* or rheumatolog* or urolog*))).ti,bt,kf. 98141

2 (resident or residents or residency).ti,bt,kf. and ("residen* education" or "residen* training" or "fellowship training" or (residen* adj3 (intern or interns or internship*)) or PGE or PGME or ((resident or residents or residency or residencies or intern or interns or internship* or fellow* or graduate or postgraduate or post-graduate) adj6 (medical or medicine or surgical or surgery or anesthesiolog* or anaesthesiolog* or cardiology or dermatolog* or endocrinology or gastroenterolog* or "general practice" or gerontolog* or gynaecolog* or gynecolog* or haematolog* or hematolog* or hepatolog* or immunolog* or neonatolog* or nephrolog* or neurolog* or neurosurg* or obstetric* or ophthalmolog* or oncolog* or orthopedic* or orthopaedic* or otolaryngolog* or otorhinolaryngolog* or paediatric* or pediatric* or pathology or podiatry or physiatry or psychiatr* or pulmonolog* or radiolog* or rheumatolog* or urolog*))).mp. 40509

3 ("academic faculty" or "academic staff" or "teaching staff" or "research faculty" or "diverse faculty" or "minority faculty" or "junior faculty" or "faculty members" or "female faculty" or "male faculty" or professor* or tenure-track or tenured or "department* chair*").ti,bt,kf. and (medical or medicine or surgical or surgery or anesthesiolog* or anaesthesiolog* or cardiology or dermatolog* or endocrinology or gastroenterolog* or "general practice" or gerontolog* or gynaecolog* or gynecolog* or haematolog* or hematolog* or hepatolog* or immunolog* or neonatolog* or nephrolog* or neurolog* or neurosurg* or obstetric* or ophthalmolog* or oncolog* or orthopedic* or orthopaedic* or otolaryngolog* or otorhinolaryngolog* or paediatric* or pediatric* or pathology or podiatry or physiatry or psychiatr* or pulmonolog* or radiolog* or rheumatolog* or urolog*).mp. 7119

4 exp *physicians/ or (physician* or "general practitioner*" or GP or doctor* or surgeon* or clinician* or internist* or specialist* or anaesthetist* or anesthesiologist* or cardiologist* or dermatologist* or diabetologist* or endocrinologist* or gastroenterologist* or geriatrician* or gerontologist* or gynaecologist* or gynecologist* or haematologist* or hematologist* or hepatologist* or immunologist* or neonatologist* or nephrologist* or neurologist* or neurosurgeon* or obstetrician* or ophthalmologist* or oncologist* or orthopedist* or otolaryngologist* or paediatrician* or pediatrician* or pathologist* or podiatrist* or physiatrist* or psychiatrist* or "primary care provider*" or "primary care practitioner*" or proctologist* or pulmonologist* or radiologist* or rheumatologist* or urologist*).ti,bt,kf. 403823

5 ("medical education" or medical or medicine or surgical or surgery or anesthesiolog* or anaesthesiolog* or cardiology or dermatolog* or endocrinology or gastroenterolog* or "general practice" or gerontolog* or gynaecolog* or gynecolog* or haematolog* or hematolog* or hepatolog* or immunolog* or neonatolog* or nephrolog* or neurolog* or neurosurg* or obstetric* or ophthalmolog* or oncolog* or orthopedic* or orthopaedic* or otolaryngolog* or otorhinolaryngolog* or paediatric* or pediatric* or pathology or podiatry or physiatry or psychiatr* or pulmonolog* or radiolog* or rheumatolog* or urolog*).ti,bt. and (resident or residents or residency or residencies or intern or interns or internship* or fellow* or graduate or postgraduate or post-graduate or physician* or "general practitioner*" or GP or doctor* or surgeon* or clinician* or internist* or specialist* or anaesthetist* or anesthesiologist* or cardiologist* or dermatologist* or diabetologist* or endocrinologist* or gastroenterologist* or geriatrician* or gerontologist* or gynaecologist* or gynecologist* or haematologist* or hematologist* or hepatologist* or immunologist* or neonatologist* or nephrologist* or neurologist* or neurosurgeon* or obstetrician* or ophthalmologist* or oncologist* or orthopedist* or otolaryngologist* or paediatrician* or pediatrician* or pathologist* or podiatrist* or physiatrist* or psychiatrist* or "primary care provider*" or "primary care practitioner*" or proctologist* or pulmonologist* or radiologist* or rheumatologist* or urologist* or "academic faculty" or "academic staff" or "teaching staff" or "research faculty" or "diverse faculty" or "minority faculty" or "junior faculty" or "faculty members" or "female faculty" or "male faculty" or professor* or tenure-track or tenured or "department* chair*" or "clinical faculty" or "clinical instructor*" or "clinical preceptor*" or "medical faculty" or "surgical faculty").mp. 436840

6 1 or 2 or 3 or 4 or 5 752927

7 exp Gender Identity/ or Transsexualism/ 28798

8 ((intersectional* adj20 (sex* or gender)) or (intersecting adj8 (sex* or gender)) or transgender* or trans-gender* or non-binary or nonbinary or "gender role*" or "gender fluid*" or genderfluid or "genderqueer" or "gender queer" or "gender divers*" or "gender minorit*" or "gender identit*" or Two-Spirit* or transexual* or trans-sex* or (trans adj3 (identit* or woman or man or person or individual or physician* or resident*))).mp. 57584

9 ("sex specific" or sex-based or sex-related or "role of sex" or sex-dependen* or gendered or "gender specific" or gender-factor* or gender-based or gender-related or gender-linked or gender-dichotom* or "role of gender" or "gender norms" or gender-dependen* or sexism or "gender bias*" or "sex* bias*").mp. 103659

10 (male or males or female* or women or men or sex or gender).ti,bt. 825184

11 (male or males or female* or women or men).ab. /freq=4 704296

12 (((male or males) adj10 female*) or (women adj10 men)).ab. 861693

13 (gender-role* or sex-role*).mp. 6659

14 ((disparit* or differen* or discrepanc*) adj5 (sex or sexes or gender* or male or males or female* or women or men)).mp. 433423

15 7 or 8 or 9 or 10 or 11 or 12 or 13 or 14 1954793

16 exp american native continental ancestry group/ or Minority groups/ or Minority health/ or Prejudice/ or Racism/ or Xenophobia/ or exp Race Relations/ or exp Ethnic Groups/ or exp Continental Population Groups/ 257438

17 ((Native* adj1 (American* or Canadian* or Alaska*)) or (Natives not "digital natives") or Tribes or Indigenous or Aborigin* or Inuit* or Inuk or Inupiat* or First Nation or First Nations or Metis or Eskimo* or Aleut* or Amerindian* or (Indian* adj3 America*) or Canadian Indian* or first people* or autochthonous people* or Torres strait islander* or Maori*).mp. 104672

18 (racialized or racism or "racial minorit*" or xenophob* or black or blacks or hispanic* or latino* or latina* or latinx or asian* or african or non-white or "people of colour" or "people of color" or ethnicity or "ethnic group*" or "ethnic minorit*" or "visible minorit*" or race or racial or minorities or "minority group*" or "minority population*" or bipoc or "english as a second language" or "foreign language" or "language other than" or "international medical graduate*" or underrepresented or under-represented).mp. 906723

19 16 or 17 or 18 1004163

20 bias*.ti,bt. or (stigma or prejudi* or xenophobi* or racism or sexism or (abuse not "child abuse") or harassment or "discriminated against" or discrimination or discriminatory or (bias* adj3 (sex or gender or race or racism)) or intersectional* or (intersecting adj6 (race or racial or gender or sex)) or microaggression* or macroaggression*).mp. 502996

21 6 and 15 and 19 and 20 1410

22 21 not ("minority patient*" or "diverse patient*" or "transgender* patient*" or "LGB* patient*" or "LGB* individual*" or "LGB* adult*" or "LGB* people" or "queer patient*" or "queer individual*" or "queer adult*" or "queer people" or "anti-fat bias*" or "weight bias").ti,bt. 1385

23 limit 22 to english language 1332

**Embase <1974 to 2025 May 01>(OVID interface)**

Date searched: May 2, 2025

Results: 1018

1 *resident/ or *residency education/ or ("residen* education" or "residen* training" or "fellowship training" or (residen* adj3 (intern or interns or internship*)) or PGE or PGME or "clinical faculty" or "clinical instructor*" or "clinical preceptor*" or "medical faculty" or "surgical faculty" or ((resident or residents or residency or residencies or intern or interns or internship* or fellow* or graduate or postgraduate or post-graduate or "academic faculty" or "academic staff" or "teaching staff" or "research faculty" or "diverse faculty" or "minority faculty" or "junior faculty" or "faculty members" or "female faculty" or "male faculty" or professor* or tenure-track or tenured or "department* chair*") and (medical or medicine or surgical or surgery or anesthesiolog* or anaesthesiolog* or cardiology or dermatolog* or endocrinology or gastroenterolog* or "general practice" or gerontolog* or gynaecolog* or gynecolog* or haematolog* or hematolog* or hepatolog* or immunolog* or neonatolog* or nephrolog* or neurolog* or neurosurg* or obstetric* or ophthalmolog* or oncolog* or orthopedic* or orthopaedic* or otolaryngolog* or otorhinolaryngolog* or paediatric* or pediatric* or pathology or podiatry or physiatry or psychiatr* or pulmonolog* or radiolog* or rheumatolog* or urolog*))).ti,bt,kf. 75641

2 (resident or residents or residency).ti,bt,kf. and ("residen* education" or "residen* training" or "fellowship training" or (residen* adj3 (intern or interns or internship*)) or PGE or PGME or ((resident or residents or residency or residencies or intern or interns or internship* or fellow* or graduate or postgraduate or post-graduate) adj6 (medical or medicine or surgical or surgery or anesthesiolog* or anaesthesiolog* or cardiology or dermatolog* or endocrinology or gastroenterolog* or "general practice" or gerontolog* or gynaecolog* or gynecolog* or haematolog* or hematolog* or hepatolog* or immunolog* or neonatolog* or nephrolog* or neurolog* or neurosurg* or obstetric* or ophthalmolog* or oncolog* or orthopedic* or orthopaedic* or otolaryngolog* or otorhinolaryngolog* or paediatric* or pediatric* or pathology or podiatry or physiatry or psychiatr* or pulmonolog* or radiolog* or rheumatolog* or urolog*))).mp. 46701

3 ("academic faculty" or "academic staff" or "teaching staff" or "research faculty" or "diverse faculty" or "minority faculty" or "junior faculty" or "faculty members" or "female faculty" or "male faculty" or professor* or tenure-track or tenured or "department* chair*").ti,bt,kf. and (medical or medicine or surgical or surgery or anesthesiolog* or anaesthesiolog* or cardiology or dermatolog* or endocrinology or gastroenterolog* or "general practice" or gerontolog* or gynaecolog* or gynecolog* or haematolog* or hematolog* or hepatolog* or immunolog* or neonatolog* or nephrolog* or neurolog* or neurosurg* or obstetric* or ophthalmolog* or oncolog* or orthopedic* or orthopaedic* or otolaryngolog* or otorhinolaryngolog* or paediatric* or pediatric* or pathology or podiatry or physiatry or psychiatr* or pulmonolog* or radiolog* or rheumatolog* or urolog*).mp. 6224

4 exp *physician/ 221854

5 (physician* or "general practitioner*" or GP or doctor* or surgeon* or clinician* or internist* or specialist* or anaesthetist* or anesthesiologist* or cardiologist* or dermatologist* or diabetologist* or endocrinologist* or gastroenterologist* or geriatrician* or gerontologist* or gynaecologist* or gynecologist* or haematologist* or hematologist* or hepatologist* or immunologist* or neonatologist* or nephrologist* or neurologist* or neurosurgeon* or obstetrician* or ophthalmologist* or oncologist* or orthopedist* or otolaryngologist* or paediatrician* or pediatrician* or pathologist* or podiatrist* or physiatrist* or psychiatrist* or "primary care provider*" or "primary care practitioner*" or proctologist* or pulmonologist* or radiologist* or rheumatologist* or urologist*).ti,bt,kf. 405191

6 ("medical education" or medical or medicine or surgical or surgery or anesthesiolog* or anaesthesiolog* or cardiology or dermatolog* or endocrinology or gastroenterolog* or "general practice" or gerontolog* or gynaecolog* or gynecolog* or haematolog* or hematolog* or hepatolog* or immunolog* or neonatolog* or nephrolog* or neurolog* or neurosurg* or obstetric* or ophthalmolog* or oncolog* or orthopedic* or orthopaedic* or otolaryngolog* or otorhinolaryngolog* or paediatric* or pediatric* or pathology or podiatry or physiatry or psychiatr* or pulmonolog* or radiolog* or rheumatolog* or urolog*).ti,bt. and (resident or residents or residency or residencies or intern or interns or internship* or fellow* or graduate or postgraduate or post-graduate or physician* or "general practitioner*" or GP or doctor* or surgeon* or clinician* or internist* or specialist* or anaesthetist* or anesthesiologist* or cardiologist* or dermatologist* or diabetologist* or endocrinologist* or gastroenterologist* or geriatrician* or gerontologist* or gynaecologist* or gynecologist* or haematologist* or hematologist* or hepatologist* or immunologist* or neonatologist* or nephrologist* or neurologist* or neurosurgeon* or obstetrician* or ophthalmologist* or oncologist* or orthopedist* or otolaryngologist* or paediatrician* or pediatrician* or pathologist* or podiatrist* or physiatrist* or psychiatrist* or "primary care provider*" or "primary care practitioner*" or proctologist* or pulmonologist* or radiologist* or rheumatologist* or urologist* or "academic faculty" or "academic staff" or "teaching staff" or "research faculty" or "diverse faculty" or "minority faculty" or "junior faculty" or "faculty members" or "female faculty" or "male faculty" or professor* or tenure-track or tenured or "department* chair*" or "clinical faculty" or "clinical instructor*" or "clinical preceptor*" or "medical faculty" or "surgical faculty").mp. 619507

7 1 or 2 or 4 or 5 or 6 982102

8 gender identity/ or *"gender and sex"/ or transgenderism/ 25803

9 ((intersectional* adj20 (sex* or gender)) or (intersecting adj8 (sex* or gender)) or transgender* or trans-gender* or non-binary or nonbinary or "gender role*" or "gender fluid*" or genderfluid or "genderqueer" or "gender queer" or "gender divers*" or "gender minorit*" or "gender identit*" or Two-Spirit* or transexual* or trans-sex* or (trans adj3 (identit* or woman or man or person or individual or physician* or resident*))).mp. 62407

10 ("sex specific" or sex-based or sex-related or "role of sex" or sex-dependen* or gendered or "gender specific" or gender-factor* or gender-based or gender-related or gender-linked or gender-dichotom* or "role of gender" or "gender norms" or gender-dependen* or sexism or "gender bias*" or "sex* bias*").mp. 132767

11 (male or males or female* or women or men or sex or gender).ti,bt. 996177

12 (male or males or female* or women or men).ab. /freq=4 988108

13 (((male or males) adj10 female*) or (women adj10 men)).ab. 1297689

14 (gender-role* or sex-role*).mp. 10014

15 ((disparit* or differen* or discrepanc*) adj5 (sex or sexes or gender* or male or males or female* or women or men)).mp. 739605

16 8 or 9 or 10 or 11 or 12 or 13 or 14 or 15 2720220

17 ancestry group/ or asian american/ or exp black person/ or british asian/ or exp hispanic/ or exp indigenous people/ or exp oceanic ancestry group/ or exp "people of mixed ancestry"/ 310648

18 minority group/ or "people of color"/ 20454

19 racism/ 16598

20 ethnic group/ 87294

21 ((Native* adj1 (American* or Canadian* or Alaska*)) or (Natives not "digital natives") or Tribes or Indigenous or Aborigin* or Inuit* or Inuk or Inupiat* or First Nation or First Nations or Metis or Eskimo* or Aleut* or Amerindian* or (Indian* adj3 America*) or Canadian Indian* or first people* or autochthonous people* or Torres strait islander* or Maori*).mp. 125773

22 (racialized or racism or "racial minorit*" or xenophob* or black or blacks or hispanic* or latino* or latina* or latinx or asian* or african or non-white or "people of colour" or "people of color" or ethnicity or "ethnic group*" or "ethnic minorit*" or "visible minorit*" or race or racial or minorities or "minority group*" or "minority population*" or bipoc or "english as a second language" or "foreign language" or "language other than" or "international medical graduate*" or underrepresented or under-represented).mp. 1231461

23 17 or 18 or 19 or 20 or 21 or 22 1319220

24 bias*.ti,bt. or (stigma or prejudi* or xenophobi* or racism or sexism or (abuse not "child abuse") or harassment or "discriminated against" or discrimination or discriminatory or (bias* adj3 (sex or gender or race or racism)) or intersectional* or (intersecting adj6 (race or racial or gender or sex)) or microaggression* or macroaggression*).mp. 692668

25 7 and 16 and 23 and 24 1361

26 25 not ("minority patient*" or "diverse patient*" or "transgender* patient*" or "LGB* patient*" or "LGB* individual*" or "LGB* adult*" or "LGB* people" or "queer patient*" or "queer individual*" or "queer adult*" or "queer people" or "anti-fat bias*" or "weight bias").ti,bt. 1345

27 limit 26 to conference abstracts 322

28 26 not 27 1023

29 limit 28 to english language 1018

**APA PsycInfo <1806 to April 2025 Week 4>**

Date searched: May 2, 2025

Results: 450

1 *medical internship/ or *medical residency/ or ("residen* education" or "residen* training" or "fellowship training" or (residen* adj3 (intern or interns or internship*)) or PGE or PGME or "clinical faculty" or "clinical instructor*" or "clinical preceptor*" or "medical faculty" or "surgical faculty" or ((resident or residents or residency or residencies or intern or interns or internship* or fellow* or graduate or postgraduate or post-graduate or "academic faculty" or "academic staff" or "teaching staff" or "research faculty" or "diverse faculty" or "minority faculty" or "junior faculty" or "faculty members" or "female faculty" or "male faculty" or professor* or tenure-track or tenured or "department chair*") and (medical or medicine or surgical or surgery or anesthesiolog* or anaesthesiolog* or cardiology or dermatolog* or endocrinology or gastroenterolog* or "general practice" or gerontolog* or gynaecolog* or gynecolog* or haematolog* or hematolog* or hepatolog* or immunolog* or neonatolog* or nephrolog* or neurolog* or neurosurg* or obstetric* or ophthalmolog* or oncolog* or orthopedic* or orthopaedic* or otolaryngolog* or otorhinolaryngolog* or paediatric* or pediatric* or pathology or podiatry or physiatry or psychiatr* or pulmonolog* or radiolog* or rheumatolog* or urolog*))).ti,bt,id. 10983

2 (resident or residents or residency).ti,bt,id. and ("residen* education" or "residen* training" or "fellowship training" or (residen* adj3 (intern or interns or internship*)) or PGE or PGME or ((resident or residents or residency or residencies or intern or interns or internship* or fellow* or graduate or postgraduate or post-graduate) adj6 (medical or medicine or surgical or surgery or anesthesiolog* or anaesthesiolog* or cardiology or dermatolog* or endocrinology or gastroenterolog* or "general practice" or gerontolog* or gynaecolog* or gynecolog* or haematolog* or hematolog* or hepatolog* or immunolog* or neonatolog* or nephrolog* or neurolog* or neurosurg* or obstetric* or ophthalmolog* or oncolog* or orthopedic* or orthopaedic* or otolaryngolog* or otorhinolaryngolog* or paediatric* or pediatric* or pathology or podiatry or physiatry or psychiatr* or pulmonolog* or radiolog* or rheumatolog* or urolog*))).mp. 7209

3 ("academic faculty" or "academic staff" or "teaching staff" or "research faculty" or "diverse faculty" or "minority faculty" or "junior faculty" or "faculty members" or "female faculty" or "male faculty" or professor* or tenure-track or tenured or "department* chair*").ti,bt,id. and (medical or medicine or surgical or surgery or anesthesiolog* or anaesthesiolog* or cardiology or dermatolog* or endocrinology or gastroenterolog* or "general practice" or gerontolog* or gynaecolog* or gynecolog* or haematolog* or hematolog* or hepatolog* or immunolog* or neonatolog* or nephrolog* or neurolog* or neurosurg* or obstetric* or ophthalmolog* or oncolog* or orthopedic* or orthopaedic* or otolaryngolog* or otorhinolaryngolog* or paediatric* or pediatric* or pathology or podiatry or physiatry or psychiatr* or pulmonolog* or radiolog* or rheumatolog* or urolog*).mp. 887

4 exp *physicians/ or (physician* or "general practitioner*" or GP or doctor* or surgeon* or clinician* or internist* or specialist* or anaesthetist* or anesthesiologist* or cardiologist* or dermatologist* or diabetologist* or endocrinologist* or gastroenterologist* or geriatrician* or gerontologist* or gynaecologist* or gynecologist* or haematologist* or hematologist* or hepatologist* or immunologist* or neonatologist* or nephrologist* or neurologist* or neurosurgeon* or obstetrician* or ophthalmologist* or oncologist* or orthopedist* or otolaryngologist* or paediatrician* or pediatrician* or pathologist* or podiatrist* or physiatrist* or psychiatrist* or "primary care provider*" or "primary care practitioner*" or proctologist* or pulmonologist* or radiologist* or rheumatologist* or urologist*).ti,bt,id. 89673

5 ("medical education" or medical or medicine or surgical or surgery or anesthesiolog* or anaesthesiolog* or cardiology or dermatolog* or endocrinology or gastroenterolog* or "general practice" or gerontolog* or gynaecolog* or gynecolog* or haematolog* or hematolog* or hepatolog* or immunolog* or neonatolog* or nephrolog* or neurolog* or neurosurg* or obstetric* or ophthalmolog* or oncolog* or orthopedic* or orthopaedic* or otolaryngolog* or otorhinolaryngolog* or paediatric* or pediatric* or pathology or podiatry or physiatry or psychiatr* or pulmonolog* or radiolog* or rheumatolog* or urolog*).ti,bt. and (resident or residents or residency or residencies or intern or interns or internship* or fellow* or graduate or postgraduate or post-graduate or physician* or "general practitioner*" or GP or doctor* or surgeon* or clinician* or internist* or specialist* or anaesthetist* or anesthesiologist* or cardiologist* or dermatologist* or diabetologist* or endocrinologist* or gastroenterologist* or geriatrician* or gerontologist* or gynaecologist* or gynecologist* or haematologist* or hematologist* or hepatologist* or immunologist* or neonatologist* or nephrologist* or neurologist* or neurosurgeon* or obstetrician* or ophthalmologist* or oncologist* or orthopedist* or otolaryngologist* or paediatrician* or pediatrician* or pathologist* or podiatrist* or physiatrist* or psychiatrist* or "primary care provider*" or "primary care practitioner*" or proctologist* or pulmonologist* or radiologist* or rheumatologist* or urologist* or "academic faculty" or "academic staff" or "teaching staff" or "research faculty" or "diverse faculty" or "minority faculty" or "junior faculty" or "faculty members" or "female faculty" or "male faculty" or professor* or tenure-track or tenured or "department chair*" or "clinical faculty" or "clinical instructor*" or "clinical preceptor*" or "medical faculty" or "surgical faculty").mp. 65364

6 1 or 2 or 3 or 4 or 5 135201

7 exp gender identity/ or gender diversity/ or transgender/ or transsexualism/ 54583

8 ((intersectional* adj20 (sex* or gender)) or (intersecting adj8 (sex* or gender)) or transgender* or trans-gender* or non-binary or nonbinary or "gender role*" or "gender fluid*" or genderfluid or "genderqueer" or "gender queer" or "gender divers*" or "gender minorit*" or "gender identit*" or Two-Spirit* or transexual* or trans-sex* or (trans adj3 (identit* or woman or man or person or individual or physician* or resident*))).mp. 57933

9 ("sex specific" or sex-based or sex-related or "role of sex" or sex-dependen* or gendered or "gender specific" or gender-factor* or gender-based or gender-related or gender-linked or gender-dichotom* or "role of gender" or "gender norms" or gender-dependen* or sexism or "gender bias*" or "sex* bias*").mp. 57452

10 (male or males or female* or women or men or sex or gender).ti,bt. 308161

11 (male or males or female* or women or men).ab. /freq=4 232691

12 (((male or males) adj10 female*) or (women adj10 men)).ab. 284497

13 (gender-role* or sex-role*).mp. 39277

14 ((disparit* or differen* or discrepanc*) adj5 (sex or sexes or gender* or male or males or female* or women or men)).mp. 224539

15 7 or 8 or 9 or 10 or 11 or 12 or 13 or 14 660250

16 "racial and ethnic groups"/ or african cultural groups/ or exp asians/ or black people/ or exp indigenous populations/ or exp "latinos/latinas"/ or exp "middle eastern and north african cultural groups"/ or multiracial/ or "people of color"/ or south american cultural groups/ or ethnic bias/ or exp minority groups/ or "racial and ethnic differences"/ or racial bias/ or racial disparities/ or racial diversity/ or racial identity/ or racial microaggression/ or racial trauma/ or systemic racism/ 211816

17 ((Native* adj1 (American* or Canadian* or Alaska*)) or (Natives not "digital natives") or Tribes or Indigenous or Aborigin* or Inuit* or Inuk or Inupiat* or First Nation or First Nations or Metis or Eskimo* or Aleut* or Amerindian* or (Indian* adj3 America*) or Canadian Indian* or first people* or autochthonous people* or Torres strait islander* or Maori*).mp. 41058

18 (racialized or racism or "racial minorit*" or xenophob* or black or blacks or hispanic* or latino* or latina* or latinx or asian* or african or non-white or "people of colour" or "people of color" or ethnicity or "ethnic group*" or "ethnic minorit*" or "visible minorit*" or race or racial or minorities or "minority group*" or "minority population*" or bipoc or "english as a second language" or "foreign language" or "language other than" or "international medical graduate*" or underrepresented or under-represented).mp. 420373

19 16 or 17 or 18 465068

20 bias*.ti,bt. or (stigma or prejudi* or xenophobi* or racism or sexism or (abuse not "child abuse") or harassment or "discriminated against" or discrimination or discriminatory or (bias* adj3 (sex or gender or race or racism)) or intersectional* or (intersecting adj6 (race or racial or gender or sex)) or microaggression* or macroaggression*).mp. 410284

21 6 and 15 and 19 and 20 465

22 21 not ("minority patient*" or "diverse patient*" or "transgender* patient*" or "LGB* patient*" or "LGB* individual*" or "LGB* adult*" or "LGB* people" or "queer patient*" or "queer individual*" or "queer adult*" or "queer people" or "anti-fat bias*" or "weight bias").ti,bt. 458

23 limit 22 to english language 450

**CINAHL Plus with Full Text (EBSCOhost interface)**

Date searched: May 2, 2025

Results: 397

S1 (MM "Interns and Residents") OR (MM "Internship and Residency") OR (MM "Medical Fellowships") OR (MM "Faculty, Medical") OR (MM "Physicians+") OR TI(("residen* education" or "residen* training" or "fellowship training" or (residen* N3 (intern or interns or internship*)) or PGE or PGME or "clinical faculty" or "clinical instructor*" or "clinical preceptor*" or "medical faculty" or "surgical faculty" or ((resident or residents or residency or residencies or intern or interns or internship* or fellow* or graduate or postgraduate or post-graduate or "academic faculty" or "academic staff" or "teaching staff" or "research faculty" or "diverse faculty" or "minority faculty" or "junior faculty" or "faculty members" or "female faculty" or "male faculty" or professor* or tenure-track or tenured or "department chair*") and (medical or medicine or surgical or surgery or anesthesiolog* or anaesthesiolog* or cardiology or dermatolog* or endocrinology or gastroenterolog* or "general practice" or gerontolog* or gynaecolog* or gynecolog* or haematolog* or hematolog* or hepatolog* or immunolog* or neonatolog* or nephrolog* or neurolog* or neurosurg* or obstetric* or ophthalmolog* or oncolog* or orthopedic* or orthopaedic* or otolaryngolog* or otorhinolaryngolog* or paediatric* or pediatric* or pathology or podiatry or physiatry or psychiatr* or pulmonolog* or radiolog* or rheumatolog* or urolog*)))

S2 TI(resident or residents or residency) AND ("residen* education" or "residen* training" or "fellowship training" or (residen* N3 (intern or interns or internship*)) or PGE or PGME or ((resident or residents or residency or residencies or intern or interns or internship* or fellow* or graduate or postgraduate or post-graduate) N6 (medical or medicine or surgical or surgery or anesthesiolog* or anaesthesiolog* or cardiology or dermatolog* or endocrinology or gastroenterolog* or "general practice" or gerontolog* or gynaecolog* or gynecolog* or haematolog* or hematolog* or hepatolog* or immunolog* or neonatolog* or nephrolog* or neurolog* or neurosurg* or obstetric* or ophthalmolog* or oncolog* or orthopedic* or orthopaedic* or otolaryngolog* or otorhinolaryngolog* or paediatric* or pediatric* or pathology or podiatry or physiatry or psychiatr* or pulmonolog* or radiolog* or rheumatolog* or urolog*)))

S3 TI("academic faculty" or "academic staff" or "teaching staff" or "research faculty" or "diverse faculty" or "minority faculty" or "junior faculty" or "faculty members" or "female faculty" or "male faculty" or professor* or tenure-track or tenured or "department* chair*") AND (medical or medicine or surgical or surgery or anesthesiolog* or anaesthesiolog* or cardiology or dermatolog* or endocrinology or gastroenterolog* or "general practice" or gerontolog* or gynaecolog* or gynecolog* or haematolog* or hematolog* or hepatolog* or immunolog* or neonatolog* or nephrolog* or neurolog* or neurosurg* or obstetric* or ophthalmolog* or oncolog* or orthopedic* or orthopaedic* or otolaryngolog* or otorhinolaryngolog* or paediatric* or pediatric* or pathology or podiatry or physiatry or psychiatr* or pulmonolog* or radiolog* or rheumatolog* or urolog*)

S4 TI(physician* or "general practitioner*" or GP or doctor* or surgeon* or clinician* or internist* or specialist* or anaesthetist* or anesthesiologist* or cardiologist* or dermatologist* or diabetologist* or endocrinologist* or gastroenterologist* or geriatrician* or gerontologist* or gynaecologist* or gynecologist* or haematologist* or hematologist* or hepatologist* or immunologist* or neonatologist* or nephrologist* or neurologist* or neurosurgeon* or obstetrician* or ophthalmologist* or oncologist* or orthopedist* or otolaryngologist* or paediatrician* or pediatrician* or pathologist* or podiatrist* or physiatrist* or psychiatrist* or "primary care provider*" or "primary care practitioner*" or proctologist* or pulmonologist* or radiologist* or rheumatologist* or urologist*)

S5 TI("medical education" or medical or medicine or surgical or surgery or anesthesiolog* or anaesthesiolog* or cardiology or dermatolog* or endocrinology or gastroenterolog* or "general practice" or gerontolog* or gynaecolog* or gynecolog* or haematolog* or hematolog* or hepatolog* or immunolog* or neonatolog* or nephrolog* or neurolog* or neurosurg* or obstetric* or ophthalmolog* or oncolog* or orthopedic* or orthopaedic* or otolaryngolog* or otorhinolaryngolog* or paediatric* or pediatric* or pathology or podiatry or physiatry or psychiatr* or pulmonolog* or radiolog* or rheumatolog* or urolog*) AND (resident or residents or residency or residencies or intern or interns or internship* or fellow* or graduate or postgraduate or post-graduate or physician* or "general practitioner*" or GP or doctor* or surgeon* or clinician* or internist* or specialist* or anaesthetist* or anesthesiologist* or cardiologist* or dermatologist* or diabetologist* or endocrinologist* or gastroenterologist* or geriatrician* or gerontologist* or gynaecologist* or gynecologist* or haematologist* or hematologist* or hepatologist* or immunologist* or neonatologist* or nephrologist* or neurologist* or neurosurgeon* or obstetrician* or ophthalmologist* or oncologist* or orthopedist* or otolaryngologist* or paediatrician* or pediatrician* or pathologist* or podiatrist* or physiatrist* or psychiatrist* or "primary care provider*" or "primary care practitioner*" or proctologist* or pulmonologist* or radiologist* or rheumatologist* or urologist* or "academic faculty" or "academic staff" or "teaching staff" or "research faculty" or "diverse faculty" or "minority faculty" or "junior faculty" or "faculty members" or "female faculty" or "male faculty" or professor* or tenure-track or tenured or "department chair*" or "clinical faculty" or "clinical instructor*" or "clinical preceptor*" or "medical faculty" or "surgical faculty")

S6 S1 or S2 or S3 or S4 or S5 (295,922)

S7 ( (MH "Gender Identity+") OR (MH "Transgender Persons+") ) OR ( ((intersectional* N20 (sex* or gender)) or (intersecting N8 (sex* or gender)) or transgender* or trans-gender* or non-binary or nonbinary or "gender role*" or "gender fluid*" or genderfluid or "genderqueer" or "gender queer" or "gender divers*" or "gender minorit*" or "gender identit*" or Two-Spirit* or transexual* or trans-sex* or (trans N3 (identit* or woman or man or person or individual or physician* or resident*)) OR "sex specific" or sex-based or sex-related or "role of sex" or sex-dependen* or gendered or "gender specific" or gender-factor* or gender-based or gender-related or gender-linked or gender-dichotom* or "role of gender" or "gender norms" or gender-dependen* or sexism or "gender bias*" or "sex* bias*" or "gender role*" or "sex role*" or ((disparit* or differen* or discrepanc*) N5 (sex or sexes or gender* or male or males or female* or women or men))) OR (((male or males) N10 female*) or (women N10 men)) ) OR ( TI(male or males or female* or women or men or sex or gender) ) (575,114)

S8 (MH "Racialization") OR (MH "Racial Equality") OR (MH "Ethnic Groups") OR (MH "Africans+") OR (MH "Arabs+") OR (MH "Asians+") OR (MH "Black Persons+") OR (MH "Caribbean Persons+") OR (MH "Indigenous Peoples+") OR (MH "Middle Eastern Persons+") OR (MH "Multiracial Persons+") OR (MH "South Americans+") OR ((Native* N1 (American* or Canadian* or Alaska*)) or (Natives NOT "digital natives") or Tribes or Indigenous or Aborigin* or Inuit* or Inuk or Inupiat* or "First Nation" or "First Nations" or Metis or Eskimo* or Aleut* or Amerindian* or (Indian* N3 America*) or "Canadian Indian*" or "first people*" or "autochthonous people*" or "Torres strait islander*" or Maori* or racialized or racism or "racial minorit*" or xenophob* or black or blacks or hispanic* or latino* or latina* or latinx or asian* or african or non-white or "people of colour" or "people of color" or ethnicity or "ethnic group*" or "ethnic minorit*" or "visible minorit*" or race or racial or minorities or "minority group*" or "minority population*" or bipoc or "english as a second language" or "foreign language" or "language other than" or "international medical graduate*" or underrepresented or under-represented)

S9 TI(bias*) or (stigma or prejudi* or xenophobi* or racism or sexism or (abuse not "child abuse") or harassment or "discriminated against" or discrimination or discriminatory or (bias* N3 (sex or gender or race or racism)) or intersectional* or (intersecting N6 (race or racial or gender or sex)) or microaggression* or macroaggression*)

S10 TI("minority patient*" or "diverse patient*" or "transgender* patient*" or "LGB* patient*" or "LGB* individual*" or "LGB* adult*" or "LGB* people" or "queer patient*" or "queer individual*" or "queer adult*" or "queer people" or "anti-fat bias*" or "weight bias")

S11 (S6 AND S7 AND S8 AND S9) NOT S10

**Scopus (Advanced search)**

Date searched: April 29, 2025

Results: 1440

( TITLE ( "residen* education" OR "residen* training" OR "fellowship training" OR ( residen* W/3 ( intern OR interns OR internship* ) ) OR pge OR pgme OR "clinical faculty" OR "clinical instructor*" OR "clinical preceptor*" OR "medical faculty" OR "surgical faculty" OR ( ( resident OR residents OR residency OR residencies OR intern OR interns OR internship* OR fellow* OR graduate OR postgraduate OR post-graduate OR "academic faculty" OR "academic staff" OR "teaching staff" OR "research faculty" OR "diverse faculty" OR "minority faculty" OR "junior faculty" OR "faculty members" OR "female faculty" OR "male faculty" OR professor* OR tenure-track OR tenured OR "department* chair*" ) AND ( medical OR medicine OR surgical OR surgery OR anesthesiolog* OR anaesthesiolog* OR cardiology OR dermatolog* OR endocrinology OR gastroenterolog* OR "general practice" OR gerontolog* OR gynaecolog* OR gynecolog* OR haematolog* OR hematolog* OR hepatolog* OR immunolog* OR neonatolog* OR nephrolog* OR neurolog* OR neurosurg* OR obstetric* OR ophthalmolog* OR oncolog* OR orthopedic* OR orthopaedic* OR otolaryngolog* OR otorhinolaryngolog* OR paediatric* OR pediatric* OR pathology OR podiatry OR physiatry OR psychiatr* OR pulmonolog* OR radiolog* OR rheumatolog* OR urolog* ) ) ) OR ( ( TITLE ( "academic faculty" OR "academic staff" OR "teaching staff" OR "research faculty" OR "diverse faculty" OR "minority faculty" OR "junior faculty" OR "faculty members" OR "female faculty" OR "male faculty" OR professor* OR tenure-track OR tenured OR "department* chair*" ) OR AUTHKEY ( "academic faculty" OR "academic staff" OR "teaching staff" OR "research faculty" OR "diverse faculty" OR "minority faculty" OR "junior faculty" OR "faculty members" OR "female faculty" OR "male faculty" OR professor* OR tenure-track OR tenured OR "department* chair*" ) ) AND TITLE-ABS-KEY ( medical OR medicine OR surgical OR surgery OR anesthesiolog* OR anaesthesiolog* OR cardiology OR dermatolog* OR endocrinology OR gastroenterolog* OR "general practice" OR gerontolog* OR gynaecolog* OR gynecolog* OR haematolog* OR hematolog* OR hepatolog* OR immunolog* OR neonatolog* OR nephrolog* OR neurolog* OR neurosurg* OR obstetric* OR ophthalmolog* OR oncolog* OR orthopedic* OR orthopaedic* OR otolaryngolog* OR otorhinolaryngolog* OR paediatric* OR pediatric* OR pathology OR podiatry OR physiatry OR psychiatr* OR pulmonolog* OR radiolog* OR rheumatolog* OR urolog* ) ) OR ( ( TITLE ( resident OR residents OR residency ) OR AUTHKEY ( resident OR residents OR residency ) ) AND TITLE-ABS-KEY ( "residen* education" OR "residen* training" OR "fellowship training" OR ( residen* W/3 ( intern OR interns OR internship* ) ) OR pge OR pgme OR ( ( resident OR residents OR residency OR residencies OR intern OR interns OR internship* OR fellow* OR graduate OR postgraduate OR post-graduate ) W/4 ( medical OR medicine OR surgical OR surgery OR anesthesiolog* OR anaesthesiolog* OR cardiology OR dermatolog* OR endocrinology OR gastroenterolog* OR "general practice" OR gerontolog* OR gynaecolog* OR gynecolog* OR haematolog* OR hematolog* OR hepatolog* OR immunolog* OR neonatolog* OR nephrolog* OR neurolog* OR neurosurg* OR obstetric* OR ophthalmolog* OR oncolog* OR orthopedic* OR orthopaedic* OR otolaryngolog* OR otorhinolaryngolog* OR paediatric* OR pediatric* OR pathology OR podiatry OR physiatry OR psychiatr* OR pulmonolog* OR radiolog* OR rheumatolog* OR urolog* ) ) ) ) OR AUTHKEY ( "residen* education" OR "residen* training" OR "fellowship training" OR ( residen* W/3 ( intern OR interns OR internship* ) ) OR pge OR pgme OR "clinical faculty" OR "clinical instructor*" OR "clinical preceptor*" OR "medical faculty" OR "surgical faculty" OR ( ( resident OR residents OR residency OR residencies OR intern OR interns OR internship* OR fellow* OR graduate OR postgraduate OR post-graduate OR "academic faculty" OR "academic staff" OR "teaching staff" OR "research faculty" OR "diverse faculty" OR "minority faculty" OR "junior faculty" OR "faculty members" OR "female faculty" OR "male faculty" OR professor* OR tenure-track OR tenured OR "department* chair*" ) AND ( medical OR medicine OR surgical OR surgery OR anesthesiolog* OR anaesthesiolog* OR cardiology OR dermatolog* OR endocrinology OR gastroenterolog* OR "general practice" OR gerontolog* OR gynaecolog* OR gynecolog* OR haematolog* OR hematolog* OR hepatolog* OR immunolog* OR neonatolog* OR nephrolog* OR neurolog* OR neurosurg* OR obstetric* OR ophthalmolog* OR oncolog* OR orthopedic* OR orthopaedic* OR otolaryngolog* OR otorhinolaryngolog* OR paediatric* OR pediatric* OR pathology OR podiatry OR physiatry OR psychiatr* OR pulmonolog* OR radiolog* OR rheumatolog* OR urolog* ) ) ) OR TITLE ( physician* OR "general practitioner*" OR gp OR doctor* OR surgeon* OR clinician* OR internist* OR specialist* OR anaesthetist* OR anesthesiologist* OR cardiologist* OR dermatologist* OR diabetologist* OR endocrinologist* OR gastroenterologist* OR geriatrician* OR gerontologist* OR gynaecologist* OR gynecologist* OR haematologist* OR hematologist* OR hepatologist* OR immunologist* OR neonatologist* OR nephrologist* OR neurologist* OR neurosurgeon* OR obstetrician* OR ophthalmologist* OR oncologist* OR orthopedist* OR otolaryngologist* OR paediatrician* OR pediatrician* OR pathologist* OR podiatrist* OR physiatrist* OR psychiatrist* OR "primary care provider*" OR "primary care practitioner*" OR proctologist* OR pulmonologist* OR radiologist* OR rheumatologist* OR urologist* ) OR AUTHKEY ( physician* OR "general practitioner*" OR gp OR doctor* OR surgeon* OR clinician* OR internist* OR specialist* OR anaesthetist* OR anesthesiologist* OR cardiologist* OR dermatologist* OR diabetologist* OR endocrinologist* OR gastroenterologist* OR geriatrician* OR gerontologist* OR gynaecologist* OR gynecologist* OR haematologist* OR hematologist* OR hepatologist* OR immunologist* OR neonatologist* OR nephrologist* OR neurologist* OR neurosurgeon* OR obstetrician* OR ophthalmologist* OR oncologist* OR orthopedist* OR otolaryngologist* OR paediatrician* OR pediatrician* OR pathologist* OR podiatrist* OR physiatrist* OR psychiatrist* OR "primary care provider*" OR "primary care practitioner*" OR proctologist* OR pulmonologist* OR radiologist* OR rheumatologist* OR urologist* ) OR ( TITLE ( "medical education" OR medical OR medicine OR surgical OR surgery OR anesthesiolog* OR anaesthesiolog* OR cardiology OR dermatolog* OR endocrinology OR gastroenterolog* OR "general practice" OR gerontolog* OR gynaecolog* OR gynecolog* OR haematolog* OR hematolog* OR hepatolog* OR immunolog* OR neonatolog* OR nephrolog* OR neurolog* OR neurosurg* OR obstetric* OR ophthalmolog* OR oncolog* OR orthopedic* OR orthopaedic* OR otolaryngolog* OR otorhinolaryngolog* OR paediatric* OR pediatric* OR pathology OR podiatry OR physiatry OR psychiatr* OR pulmonolog* OR radiolog* OR rheumatolog* OR urolog* ) AND TITLE-ABS-KEY ( resident OR residents OR residency OR residencies OR intern OR interns OR internship* OR fellow* OR graduate OR postgraduate OR post-graduate OR physician* OR "general practitioner*" OR gp OR doctor* OR surgeon* OR clinician* OR internist* OR specialist* OR anaesthetist* OR anesthesiologist* OR cardiologist* OR dermatologist* OR diabetologist* OR endocrinologist* OR gastroenterologist* OR geriatrician* OR gerontologist* OR gynaecologist* OR gynecologist* OR haematologist* OR hematologist* OR hepatologist* OR immunologist* OR neonatologist* OR nephrologist* OR neurologist* OR neurosurgeon* OR obstetrician* OR ophthalmologist* OR oncologist* OR orthopedist* OR otolaryngologist* OR paediatrician* OR pediatrician* OR pathologist* OR podiatrist* OR physiatrist* OR psychiatrist* OR "primary care provider*" OR "primary care practitioner*" OR proctologist* OR pulmonologist* OR radiologist* OR rheumatologist* OR urologist* OR "academic faculty" OR "academic staff" OR "teaching staff" OR "research faculty" OR "diverse faculty" OR "minority faculty" OR "junior faculty" OR "faculty members" OR "female faculty" OR "male faculty" OR professor* OR tenure-track OR tenured OR "department* chair*" OR "clinical faculty" OR "clinical instructor*" OR "clinical preceptor*" OR "medical faculty" OR "surgical faculty" ) ) ) AND ( ( ( TITLE-ABS-KEY ( ( intersectional* W/20 ( sex* OR gender ) ) OR ( intersecting W/8 ( sex* OR gender ) ) OR transgender* OR trans-gender* OR non-binary OR nonbinary OR "gender role*" OR "gender fluid*" OR genderfluid OR "genderqueer" OR "gender queer" OR "gender divers*" OR "gender minorit*" OR "gender identit*" OR two-spirit* OR transexual* OR trans-sex* OR ( trans W/3 ( identit* OR woman OR man OR person OR individual OR physician* OR resident* ) ) OR "sex specific" OR sex-based OR sex-related OR "role of sex" OR sex-dependen* OR gendered OR "gender specific" OR gender-factor* OR gender-based OR gender-related OR gender-linked OR gender-dichotom* OR "role of gender" OR "gender norms" OR gender-dependen* OR sexism OR "gender bias*" OR "sex* bias*" OR gender-role* OR sex-role* OR ( ( disparit* OR differen* OR discrepanc* ) W/5 ( sex OR sexes OR gender* OR male OR males OR female* OR women OR men ) ) ) OR TITLE ( male OR males OR female* OR women OR men OR sex OR gender ) OR ABS ( ( ( male OR males ) W/10 female* ) OR ( women W/10 men ) ) ) AND TITLE-ABS-KEY ( ( native* W/1 ( american* OR canadian* OR alaska* ) ) OR tribes OR indigenous OR aborigin* OR inuit* OR inuk OR inupiat* OR "First Nation" OR "First Nations" OR metis OR eskimo* OR aleut* OR amerindian* OR ( indian* W/3 america* ) OR "Canadian Indian*" OR "first people*" OR "autochthonous people*" OR "Torres strait islander*" OR maori* OR racialized OR racism OR "racial minorit*" OR xenophob* OR black OR blacks OR hispanic* OR latino* OR latina* OR latinx OR asian* OR african OR non-white OR "people of colour" OR "people of color" OR ethnicity OR "ethnic group*" OR "ethnic minorit*" OR "visible minorit*" OR race OR racial OR minorities OR "minority group*" OR "minority population*" OR bipoc OR "english as a second language" OR "foreign language" OR "language other than" OR "international medical graduate*" OR underrepresented OR under-represented ) AND ( TITLE ( bias* ) OR TITLE-ABS-KEY ( stigma OR prejudi* OR xenophobi* OR racism OR sexism OR ( abuse AND NOT "child abuse" ) OR harassment OR "discriminated against" OR discrimination OR discriminatory OR ( bias* W/3 ( sex OR gender OR race OR racism ) ) OR intersectional* OR ( intersecting W/6 ( race OR racial OR gender OR sex ) ) OR microaggression* OR macroaggression* ) ) ) AND NOT TITLE ( "minority patient*" OR "diverse patient*" OR "transgender* patient*" OR "LGB* patient*" OR "LGB* individual*" OR "LGB* adult*" OR "LGB* people" OR "queer patient*" OR "queer individual*" OR "queer adult*" OR "queer people" OR "anti-fat bias*" OR "weight bias" ) ) AND ( LIMIT-TO ( LANGUAGE , "English" ) )
